# Speciesism in everyday language

**DOI:** 10.1111/bjso.12561

**Published:** 2022-07-30

**Authors:** Stefan Leach, Andrew P. Kitchin, Robbie M. Sutton, Kristof Dhont

**Affiliations:** ^1^ School of Psychology University of Kent Kent UK

**Keywords:** animals, human‐animal relations, natural language processing, speciesism, word embeddings

## Abstract

Speciesism, like other forms of prejudice, is thought to be underpinned by biased patterns of language use. Thus far, however, psychological science has primarily focused on how speciesism is reflected in individuals' thoughts as opposed to wider collective systems of meaning such as language. We present a large‐scale quantitative test of speciesism by applying machine‐learning methods (word embeddings) to billions of English words derived from conversation, film, books, and the Internet. We found evidence of anthropocentric speciesism: words denoting concern (vs. indifference) and value (vs. valueless) were more closely associated with words denoting humans compared to many other animals. We also found evidence of companion animal speciesism: the same words were more closely associated with words denoting companion animals compared to most other animals. The work describes speciesism as a pervasive collective phenomenon that is evident in a naturally occurring expression of human psychology – everyday language.

## BACKGROUND

The Great Chain of Being presents an anthropocentric view of the universe: a graded continuum of existence with humans above animals (Lovejoy, 1937). Although ancient, the metaphor remains applicable to modern society and is consistent with what philosophers and activists have described as a collective form of discrimination against different species. Speciesism can be defined as the disadvantageous treatment, consideration, or assignment of moral standing based on species membership (Caviola et al., 2018, 2022; Dhont et al., 2020; Horta, 2010; Ryder, 2010; Singer, 2009b). The sheer scale at which we harm animals for human benefit, such as pigs for food and mice for medicine, attests to our tendency towards *anthropocentric speciesism* (valuing humans over other animals). Likewise, the unique treatment and protections we reserve for some animals, such as dogs and cats, reflects our propensity towards *companion animal speciesism* (valuing companion animals over other animals).

### Speciesism in belief

Anthropocentric speciesism manifests in the belief that the welfare of humans is more valuable compared to, for example, dolphins, chimps, cows, and chickens (Crimston et al., 2016) and is expressed in the greater discomfort felt at the thought of harming humans compared to chimps, dogs, and frogs (Gray et al., 2007). Similar preferences emerge when the welfare of humans and animals are pitted against one another. People give more to causes that support humans compared to animals (Caviola et al., 2018). They also prefer autonomous vehicles that swerve to save a human but kill a cat or dog more than vehicles that do the opposite (Awad et al., 2018). Likewise, people are more reluctant to sacrifice a human to save several humans than they are to sacrifice a dog, chimp, or pig to save several dogs, chimps, or pigs. This suggests that they experience greater prohibitions against harming humans compared to animals (Caviola et al., 2020). These data capture the fact that humans are granted superior moral status over animals – a pattern that is consistent with anthropocentric speciesism.

Research demonstrates how companion animal speciesism manifests in belief. Dogs and cats are extended unique protections and in some cases are considered to be ‘like a person’ (Voith et al., 1992). The status of companion animals is particularly striking when contrasted with animals reared for food. The harms inflicted upon food animals are typically seen as permissible and culturally normative (Loughnan & Davies, 2020; Piazza et al., 2015), whereas treating dogs or cats in a similar way is offensive in many cultures (Haidt et al., 1993; Possidónio et al., 2019). This is backed up by diminished feelings of concern for pigs, cows, and chickens compared to dogs and cats (Krings et al., 2021; Leite et al., 2019; Possidónio et al., 2019). The privileged status of companion animals is also revealed in preference for causes that support the welfare of dogs compared to pigs (Caviola & Capraro, 2020). Other work demonstrates that people are more concerned with the welfare of companion animals compared to appealing (e.g., dolphins) and unappealing wild animals (e.g., snakes: Leite et al., 2019; Possidónio et al., 2019). These data suggest that companion animals hold a privileged status compared to other animals – a pattern that is consistent with companion animal speciesism.

### Speciesism in language

It is clear that speciesism is reflected in beliefs about the value of humans and animals. These data are important but they do not do justice to the social reality of speciesism. Speciesism describes a broader collective orientation towards animals (Dhont et al., 2020; Dunayer, 2004; Singer, 2009a, 2009b). To progress on this front and move towards an empirical account of speciesism as such, we propose to examine a pervasive psychological expression of our collective orientation towards animals – everyday language (Durkheim, 1972; Moscovici, 1988). Everyday language is an ideal medium for such an investigation because it can reveal what we care about and how we view the world (Boyd & Schwartz, 2021; Pennebaker et al., 2003). It also acts as a vehicle for social learning by transmitting information from person to person (Kashima, 2000, 2008; Lyons & Kashima, 2001, 2003). Recent work demonstrates this by showing how moral sentiments, such as ‘care’, are denoted in language (Garten et al., 2016; Graham et al., 2009; Sagi & Dehghani, 2014) and how they can propagate on a global scale via social media platforms (Brady et al., 2017). This work illustrates the importance of understanding the role that language plays in moral phenomena, and suggests that a satisfying empirical account of speciesism needs to take into account how it manifests in language.

Scholars and activists have been vocal in highlighting examples of how speciesism manifests in our collective use of language (Cole & Morgan, 2011; Dunayer, 2004; Goodall, 1990; PETA, 2021; Sealey & Oakley, 2013; Singer, 2009a; Stibbe, 2015). Joan Dunayer (2004) presents a comprehensive qualitative analysis of the often subtle ways in which we collectively demean animals. For example, downplaying their suffering by denoting it with less extreme terms. Goodall (1990) recalls the scientific community's requirement to avoid anthropomorphic language and to refer to animals as non‐moral objects, which arguably serves to reduce concern for them. In a similar vein, Peter Singer (2009a) argues that media portrayals of farmed animals, such as pigs and cows, differ from those of other animals, such as dogs and cats, and that these differences uphold the unjustified lower concern for food animals compared to, for example, companion animals. These perspectives present speciesism, both in its *anthropocentric* and *companion animal* forms, as collective representations of humans and animals that are revealed in everyday language (Durkheim, 1972; Moscovici, 1988).

Despite these arguments, there have been practically no quantitative investigations into the nature and pervasiveness of speciesism in everyday language. The availability of digital records of language as it naturally occurs in everyday life (e.g., in conversation, TV, film, books, and the Internet) presents an opportunity to test for collective representations of speciesism. This type of data are ideal because it captures how people spontaneously speak and write about humans and animals. The relative abundance of this data – existing corpora consists of billions of words – requires advanced methods to effectively quantify the information therein. Recent advances in computational linguistics, known as word embeddings, can extract statistical regularities from these corpora that reveal how different groups are represented in terms of attitudes towards them and stereotypes about them (Caliskan et al., 2017; Charlesworth et al., 2021; Garg et al., 2018; Lewis & Lupyan, 2020). When trained on sufficiently large and varied corpora, these models can provide insights into the nature of collective representations (Charlesworth et al., 2021). These methods present an opportunity to quantify how we speak and write about humans and animals and to better understand the extent to which speciesism is present in everyday language. In other words, they set the stage for a novel contribution to the psychology of speciesism by making claims about the collective and pervasive nature of the construct empirically tractable.

### Present work

We aimed to provide an account of speciesism in everyday language by quantifying collective representations of humans and animals in archives of English language derived from conversation, TV, film, books, social media, news reporting, and informational websites. We did this by analysing the associations between words denoting humans (e.g., person), companion animals (e.g., dogs), appealing wild animals (e.g., dolphins), food animals (e.g., pigs), and unappealing wild animals (e.g., snakes), and words denoting concern (e.g., care), indifference (e.g., apathy), value (e.g., important), and valueless (e.g., unimportant). The latter categories of words denote constructs that are indicative of moral standing and therefore, in this context, speciesism.

We tested two hypotheses. Hypothesis 1 derives from the idea that humans are valued more than animals (anthropocentric speciesism). This perspective entails that humans hold a privileged moral status over companion animals, appealing wild animals, food animals, and unappealing wild animals (Caviola et al., 2018; Crimston et al., 2016; Gray et al., 2007; Wilks et al., 2021); and predicts that words denoting humans will be more strongly associated with words denoting concern (vs. indifference) and value (vs. valueless) than they are with words denoting companion animals, appealing wild animals, food animals, and unappealing wild animals. Hypothesis 2 derives from the idea that companion animals are valued more than other animals (companion animal speciesism). This perspective entails that companion animals hold a privileged moral status over appealing wild animals, food animals, and unappealing wild animals (Krings et al., 2021; Leite et al., 2019; Loughnan & Davies, 2020); and predicts that words denoting companion animals will be more strongly associated with words denoting concern (vs. indifference) and value (vs. valueless) than they are with words denoting appealing wild animals, food animals, and unappealing wild animals.

## METHODS AND MATERIALS

The methods, data, and analysis script are available via the *Open Science Framework* (https://osf.io/nzsr3/).

### Primer on word embeddings

Word embeddings refer to a set of techniques that model language by analysing how words co‐occur (Mikolov, Chen, et al., 2013; Pennington et al., 2014). They do this by representing words as vectors in a high dimensional space. Vectors are often referred to as word embeddings because the words have been embedded into the vector space. The words in these models can be thought of as a cloud of points, with each point representing a word. The positions of the words quantify how they tend to co‐occur in an underlying corpus. Words that are closer together tend to co‐occur in more similar ways than words that are further apart.

Defining the positions of the words can be done via a neural network, trained over a large corpus of text to predict the occurrence of target words from their surrounding words. To train the neural network, the corpus is broken into training samples, each consisting of a target word and the surrounding words (the *n* words occurring before and after a target word). For example, if our corpus contained the string of words ‘please put the knife on the table’, we could make this into a training example by removing the word ‘knife’ and tasking the model to predict it from the surrounding words: ‘please put the ____ on the table’. The goal is to increase the probability that the model outputs the target word given the surrounding words as an input. To begin, each word in the training corpus is assigned a random position in the vector space. By iterating over the training samples, the positions of the words are shifted to optimize the predictions. The errors are minimized by checking whether the distance between words in the vector space better corresponds to how often and closely they co‐occur in the corpus of text. Through this process, the cloud of words slowly starts to organize itself, with words that tend to co‐occur in more similar ways moving closer together and words that do not moving further apart.

These models have proven to be capable of answering social questions. By analysing how words co‐occur, they are able to capture how different groups are represented in language in terms of attitudes towards them and stereotypes about them (Caliskan et al., 2017; Caliskan & Lewis, 2020; Charlesworth et al., 2021; Garg et al., 2018; Lewis & Lupyan, 2020). The reasoning here is that the geometric positions of clusters of words representing psychological and social constructs provide an indication of how these constructs are represented in language. For example, Caliskan et al. (2017) examined whether European Americans are represented more positively than African Americans, as is found by measures of mental association (Greenwald et al., 1998), by analysing the distances between words representing European Americans (e.g., Brad), African American (e.g., Kareem), pleasantness (e.g., joy), and unpleasantness (e.g., nasty). European American names were relatively closer to pleasant (vs. unpleasant) words than were African American names, suggesting that the co‐occurrence relationships between the constructs are skewed in a way that is indicative of a bias towards representing European Americans as more pleasant than African Americans (Caliskan et al., 2017). Similar results have been reported about how genders (men vs. women), interests (e.g., science vs. art), traits (e.g., competence vs. warmth), and occupations (e.g., doctor vs. dancer) are represented in language (Charlesworth et al., 2021; Garg et al., 2018; Lewis & Lupyan, 2020). These results are important because they demonstrate that word embeddings can quantify ecological expressions of human psychology reflected in everyday language.

### Data and models

We utilized pre‐trained and novel word embedding models to capture how humans and animals are represented in everyday language derived from a wide range of social products. A breakdown of the data and models are provided in Table 1. The pre‐trained models were accessed via the *Gensim* repository (https://github.com/RaRe‐Technologies/gensim‐data) and captured English language on the internet from websites such as: *Google News* (https://news.google.com/), *Wikipedia* (https://en.wikipedia.org/), and *Twitter* (https://twitter.com/; Joulin et al., 2016; Mikolov et al., 2017; Mikolov, Chen, et al., 2013; Mikolov, Sutskever, et al., 2013; Pennington et al., 2014). The novel models captured English language in speech (e.g., dyadic conversation), TV and film (e.g., Snow White and the Seven Dwarfs, Breaking Bad), and books (e.g., The Legend of Sleepy Hollow). The novel models rely heavily on a set of corpora that were recently compiled and made available by Charlesworth et al. (2021) via the *Open Science Framework* (https://osf.io/kqux5/). To train the novel models, punctuation and other auxiliary data were removed (e.g., notes about a speaker's tone). We then ran the data through the *NLTK Wordnet Lemmatizer* to reduce word tokens to their root forms (e.g., cares → care; Perkins, 2014). The lemmatized data were then fed into a fastText skip‐gram algorithm using the default settings (Joulin et al., 2016; Mikolov et al., 2017; see also https://github.com/facebookresearch/fastText).

**TABLE 1:** Summary of data and models

	Description	Source	Time period	Word tokens	Method
Pre‐trained models					
Internet (General)	English websites	UMBC WebBase corpus (Han et al., 2013)	2007	~3 billion	fastText (Joulin et al., 2016; Mikolov et al., 2017; see also https://github.com/facebookresearch/fastText/)
	English informational articles from *Wikipedia* (https://en.wikipedia.org/)	Wikimedia dump (https://dumps.wikimedia.org/)	2001–2017	~9 billion	
	English news articles from *South‐East European Times* (http://www.setimes.com/)	Statistical Machine Translation SE Times Corpus (http://statmt.org/)	2007–2016	~4 billion	
Internet (News)	English news articles from *Google News* (https://news.google.com/)	*Google* (see Mikolov, Chen, et al., 2013; Mikolov, Sutskever, et al., 2013)		~100 billion	Word2Vec ( Mikolov, Chen, et al., 2013; Mikolov, Sutskever, et al., 2013; see also https://code.google.com/archive/p/word2vec/)
Internet (Wikipedia + News)	English informational articles from *Wikipedia* (https://en.wikipedia.org/)	Wikimedia dump (https://dumps.wikimedia.org/)	2001–2014	~1.6 billion	GloVe (Pennington et al., 2014)
	English news articles from *Newswire* (https://www.newswire.com/)	English Gigaword Fifth Edition (Parker et al., 2011)	1995–2011	~4.3 billion	
Internet (Twitter)	English text from *Twitter* tweets (https://twitter.com/)	Stanford GloVe project (https://nlp.stanford.edu/projects/glove/; see also Pennington et al., 2014)		~2 billion	GloVe (Pennington et al., 2014)
Novel models					
TV and Film	English transcripts from film and TV (e.g., Snow White and the Seven Dwarfs, Doctor Who, CSI, Breaking Bad)	Transcripts Wiki (http://transcripts.wikia.com/) and Simply Scripts (https://www.simplyscripts.com/). Accessed via the *Open Science Framework* (https://osf.io/kqux5/; see also Charlesworth et al., 2021)	1938–2020	~8.75 million	fastText (Joulin et al., 2016; Mikolov et al., 2017; see also https://github.com/facebookresearch/fastText)
Books	English books (e.g., The Legend of Sleepy Hollow by Washington, Blacky the Crow by Thornton Burgess)	Project Gutenberg (https://www.gutenberg.org/). Accessed via the *Open Science Framework* (https://osf.io/kqux5/; see also Charlesworth et al., 2021)	1820–1922	~44 million	fastText (Joulin et al., 2016; Mikolov et al., 2017; see also https://github.com/facebookresearch/fastText)
Speech	English dyadic telephone conversations; ~2400 conversations; Adult speakers ages range from 17 to 68 years	Switchboard‐1 Telephone Speech Corpus (Godfrey & Holliman, 1993). Accessed via the *Open Science Framework* (https://osf.io/kqux5/; see also Charlesworth et al., 2021)	1990–1991	~3 million	fastText (Joulin et al., 2016; Mikolov et al., 2017; see also https://github.com/facebookresearch/fastText)
	English dyadic parent–child conversations; ~6500 conversations; Child speakers ages range from 0 to 12 years (*M* = 2.92 years)	Child Language Data Exchange System corpus (MacWhinney, 2000). Accessed via the *Open Science Framework* (https://osf.io/kqux5/; see also Charlesworth et al., 2021)	1970–1990	~8 million	

### Word embeddings association test (WEAT)

The Word Embeddings Association Test (WEAT; Caliskan et al., 2017) provides a method for identifying how constructs are represented in corpora by quantifying the relationships between word vectors. The WEAT is commonly used as a standardized measure of the relative association between groups of words (Caliskan et al., 2017; Charlesworth et al., 2021). For example, Charlesworth et al. (2021) utilized the WEAT to quantify the associations between words representing gender and valence (among other constructs). In the case of gender and valence, the approach entails comparing the relative association between a set of words representing genders (female = she, her, etc.; male = he, him, etc.) and a set of words representing the bipolar dimension of valence (pleasantness = fun, happy, etc. vs. unpleasantness = murder, stress, etc.). The reasoning goes that if female words are more strongly associated with words reflecting pleasantness (vs. unpleasantness) than are male words, this suggests there is a bias in favour of representing women as more pleasant than men. We follow this logic to test how humans and animals are represented in terms of constructs that are indicative of speciesism.

Speciesism can be understood as the disadvantageous treatment or consideration of those who are not classified as belonging to a certain species (Horta, 2010). In this broad sense, speciesism could manifest in language in a number of ways. It could translate to representing some species in more negative terms than others. For example, just as people feel greater warmth towards dogs than they do towards pigs, so too might they represent them in language (Sevillano & Fiske, 2016). It could also translate to representing some species as more intelligent or competent than others (Gray et al., 2007; Horta & Albersmeier, 2020; Sevillano & Fiske, 2016).^1^ Although these can be considered examples of speciesism, they do not seem to capture it in its most prototypical, unjustified, and discriminatory manifestation.

Speciesism is often discussed and studied in terms of *moral standing* (Caviola et al., 2018; Dhont et al., 2020; Singer, 2009b; but see Horta & Albersmeier, 2020). This could include: the extension of rights and considerations of fairness (Opotow, 1993), feelings of guilt in response to harm (Gray et al., 2007), charitable giving (Caviola et al., 2018; Crimston et al., 2016), and the doling out of punishment (Goodwin & Benforado, 2015). These are all, to one degree or another, manifestations of being concerned for the interests of an entity and valuing them when engaging in moral reasoning (Crimston et al., 2016). Speciesism can therefore be captured succinctly in self‐reported concern for the welfare of different species and in judgements about the relative value of one species over another (Caviola et al., 2022; Krings et al., 2021; Leite et al., 2019; Wilks et al., 2021). Following this, we chose to focus on speciesism in terms of the assignment of moral standing, operationalized as how humans and animals are represented in relation to language that denotes concern (vs. indifference) and value (vs. valueless).

Where possible, we selected words denoting humans and animals from prior research (Crimston et al., 2016). We relied heavily on (Leite et al., 2019; see also Krings et al., 2021), who document how different groups of animals are afforded moral concern. This work indicates that animal species fall into four morally relevant groups: companion animals (e.g., cats), appealing wild animals (e.g., dolphins), food animals (e.g., pigs), and unappealing wild animals (e.g., snakes). We utilized words denoting all the species studied in this work. The lists are presented in Table 2.

**TABLE 2:** Words reflecting humans and animals

Category	Words
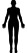 Humans	Human, humans, person, persons, peoples, people, adult, adults, teenager, teenagers, child, children, kid, kids, man, men, woman, women, lady, ladies, gentleman, gentlemen, boy, boys, girl, girls, guy, gal, baby, babies, infant, infants, toddler, toddlers
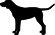 Companion Animals	Cat, cats, kitten, kittens, dog, dogs, puppy, puppies, horse, horses
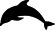 Appealing Wild Animals	Dolphin, dolphins, chimp, chimps, bear, bears, kangaroo, kangaroos
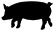 Food Animals	Chicken, chickens, chick, chicks, goat, goats, sheep, lamb, lambs, pig, pigs, turkey, turkeys, cow, cows, calf, calves, duck, ducks
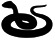 Unappealing Wild Animals	Snake, snakes, snail, snails, starfish, crocodile, crocodiles, bat, bats, frog, frogs

To our knowledge, there are no published word lists denoting concern and indifference. Because of this, we compiled lists to denote these constructs by consulting the literature on how moral concern is defined and operationalized. Moral concern can be understood as an appreciation of an entity's moral standing by virtue of its capacity to be wronged (Goodwin, 2015). The empirical literature captures moral concern in a number of ways including whether entities deserve care (Opotow, 1993) and whether entities evoke feelings of concern and sympathy (Crimston et al., 2016). We understand moral concern in similar terms, as being concerned for and caring towards an entity and as feelings of compassion and sympathy for an entity. We take indifference to be the opposite: being unconcerned and uncaring towards an entity and as feelings of apathy and disregard. On the basis of this, we compiled an initial list of ‘seed’ words (concern = *care*, *concern*, *sympathy*, *compassion*; indifference = *indifference*, *unconcerned*, *uncaring*, *apathy*, *disregard*). Following prior work (Liu, 2012), we then expanded these lists to more comprehensively capture the relevant constructs. We did this by utilizing WordNet – a comprehensive lexical database of cognitive synonyms and links between English words (Miller, 1998) – to look for words with similar meanings and senses as the initial seed words.

We are not aware of any published word lists denoting value or valueless. We compiled an initial list of seed words denoting value and valueless by consulting online dictionaries and thesauruses (value = *value*, *important*, *worth*, *significant*; valueless = *valueless*, *unimportant*, *worthless*, *insignificant*). We then expanded these lists via WordNet (Miller, 1998). The final lists denoting value and valueless are presented in Table 3.

**TABLE 3:** Words indicative of speciesism

Category	Words
Concern	Care, cares, caring, cared, concern, concerns, concerned, concerning, aid, aids, aided, aiding, help, helps, helping, helped, assist, assists, assisting, assisted, sympathy, sympathize, sympathizes, sympathized, sympathetic, sympathetically, compassion, compassions, compassionate
Indifference	Apathy, apathetic, uncaring, unaffectionate, indifference, indifferent, unconcern, unconcerned, disregard, disregards, disregarded, disregarding, detach, detaches, detaching, detached, neglect, neglects, neglected, neglecting, neglectful
Value	Value, valuable, valued, valuing, values, appreciate, appreciates, appreciated, appreciating, precious, preciously, priceless, invaluable, important, importance, importantly, worth, worthy, worthiness, significant, significantly, significance, cherish, cherished, cherishes, cherishing
Valueless	Valueless, worthless, worthlessness, insignificant, insignificantly, meritless, unimportant, unimportance, unimportantly, deficient, deficiency, insufficient, inferior, substandard, lack, lacks, lacked, lacking, disfavour, disfavours, disfavoured, disfavouring, useless, uselessness, inutility

*Note*: Vectors corresponding to some words were unavailable in some models. For a break‐down of available word vectors by model, see the Supporting Information.

We began by estimating the distance between an individual human or animal vector (e.g., person) and all the individual concern vectors (caring, concern, etc.). Following prior work, we estimated this by computing the cosine similarity between vectors (Caliskan et al., 2017; Garg et al., 2018). The resulting cosine is bounded from negative one to positive one. A cosine of one indicates the embeddings are maximally similar, whereas a cosine of negative one indicates the embeddings are maximally dissimilar. The cosine similarities (person‐caring, person‐concern, etc.) are then averaged to provide a mean association. We then did the same for the words vectors representing indifference (person‐uncaring, son‐unconcerned, etc.). Finally, we computed the difference between the two averages to provide a relative index of the strength of the association for an individual human or animal word towards concern versus indifference. We repeated this process for each human and animal word. These scores were then standardized within models as a function of their standard deviation. The whole process was then repeated for words representing value (vs. valueless).

Following prior work, we reasoned that if words denoting a group (e.g., humans) are more strongly associated with words denoting, for example, concern (vs. indifference) compared to a different group (e.g., food animals), this suggests that there is a bias in favour of representing one group as more worthy of concern than the other. We applied similar logic to words representing value (vs. valueless). Ultimately, a pattern whereby different groups are represented as more or less worthy of concern and value would be indicative of collective representations of speciesism.

To test our predictions, we planned to compare the mean WEAT scores for groups of words reflecting humans and animals. This meant that each statistical test compared two groups of words (*n* = 8–37; Table 2) derived from multiple models (*k* = 7; Table 1). Following Charlesworth et al.'s (2021) work, we assumed our results would be relatively homogenous across models (*I* = 20%). A power analysis for a random‐effects meta‐analysis via the *metapower* package for R (Version 0.2.2) suggested that these data afforded greater than 80% power (α = .050, two‐tailed) to detect effects of the following magnitudes (depending on the number of words in each group): *d* = 0.60–0.95. This seemed reasonable given the typical effect sizes reported in work utilizing word embeddings (*d* = 0.44–1.66; Caliskan et al., 2017; Charlesworth et al., 2021).

## RESULTS

### Data‐analytic approach

To provide the most reliable and generalizable findings, we estimated the overall trends across data sources and models by conducting random‐effects meta‐analyses. Effect sizes for simple comparisons were estimated from standardized mean differences (Cohen's *d*) and weighted via an inverse‐variance method (Schwarzer et al., 2015). For further model‐level estimates see the Supporting Information.

### Anthropocentric speciesism

**FIGURE 1:**
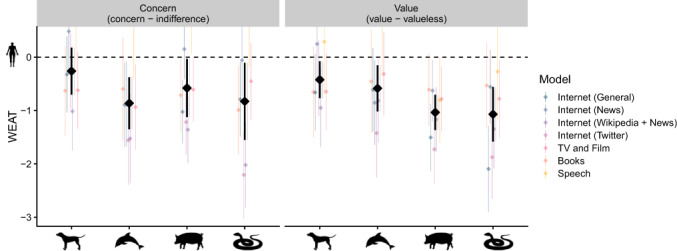
Anthropocentric speciesism in language. *Note*: WEAT is a standardized measure reflecting the average difference in the similarity between words representing humans and other groups; where y = 0 reflects no difference with humans. *Concern* reflects the difference in similarity between words denoting concern and indifference. *Value* reflects the difference in similarity between words denoting value and valueless. The figure depicts model‐level estimates (coloured diamonds), meta‐level estimates (black diamonds), and 95% CIs (whiskers)

Our first hypothesis concerns the presence of anthropocentric speciesism – that humans hold a privileged status over companion animals, appealing wild animals, food animals, and unappealing wild animals (Figure 1). Looking at words denoting concern, there was evidence of a boundary between humans and most other animals. Words denoting humans were more closely associated with words denoting concern compared to words denoting appealing wild animals, *d* = 0.86, 95% CI [0.38, 1.35], *Z* = 3.48, *p* < .001, food animals *d* = 0.58, 95% CI [0.03, 1.12], *Z* = 2.08, *p* = .038, and unappealing wild animals, *d* = 0.83, 95% CI [0.11, 1.55], *Z* = 2.25, *p* = .024. However, humans were represented in largely the same way as were companion animals, *d* = 0.26, 95% CI [−0.18, 0.70], *Z* = 1.17, *p* = .242.

Next we looked at words denoting value, where we found clear evidence of a boundary between humans and all other animals. Humans were more closely associated with value than were companion animals, *d* = 0.42, 95% CI [0.08, 0.77], *Z* = 2.41, *p* = .016, appealing wild animals, *d* = 0.59, 95% CI [0.15, 1.02], *Z* = 2.66, *p* = .008, food animals, *d* = 1.04, 95% CI [0.71, 1.37], *Z* = 6.15, *p* < .001, and unappealing wild animals, *d* = 1.07, 95% CI [0.56, 1.58], *Z* = 4.11, *p* < .001.

### Companion animal speciesism

**FIGURE 2:**
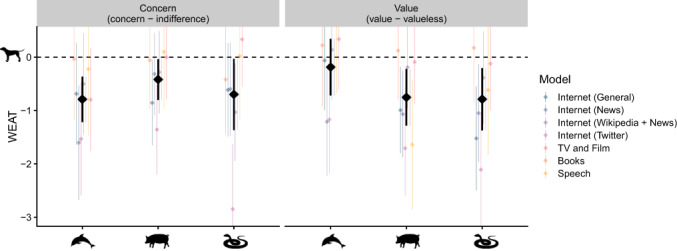
Companion animal speciesism in language. *Note*: WEAT is a standardized measure reflecting the average difference in the similarity between words representing companion animals and other groups; where y = 0 reflects no difference with companion animals. *Concern* reflects the difference in similarity between words denoting concern and indifference. *Value* reflects the difference in similarity between words denoting value and valueless. The figure depicts model‐level estimates (coloured diamonds), meta‐level estimates (black diamonds), and 95% CIs (whiskers)

Our second hypothesis concerns the presence of companion animal speciesism – that companion animals hold a privileged moral status over appealing wild animals, food animals, and unappealing wild animals (Figure 2). There was consistent evidence of companion animal speciesism when examining words denoting concern. Words denoting companion animals were more closely associated with words denoting concern compared to words denoting appealing wild animals, *d* = 0.79, 95% CI [0.37, 1.22], *Z* = 3.64, *p* < .001, food animals, *d* = 0.42, 95% CI [0.04, 0.80], *Z* = 2.15, *p* = .031, and unappealing wild animals, *d* = 0.70, 95% CI [0.03, 1.37], *Z* = 2.05, *p* = .040.

Turning to words denoting value, we also found evidence of companion animals speciesism. Words denoting companion animals were more closely associated with words denoting value (vs. valueless) than were words denoting food animals, *d* = 0.75, 95% CI [0.22, 1.28], *Z* = 2.79, *p* = .005, and unappealing wild animals, *d* = 0.79, 95% CI [0.21, 1.37], *Z* = 2.65, *p* = .008. Although we found no differences between companion animals and appealing wild animals, *d* = 0.19, 95% CI [−0.34, 0.72], *Z* = 0.69, *p* = .488.

### Additional findings

For completeness, we tested for differences between appealing wild animals, food animals, and unappealing wild animals. Looking first at words denoting concern, appealing wild animals were no different to unappealing wild animals, *d* = −0.14, 95% CI [−0.58, 0.30], *Z* = −0.62, *p* = .536, although they were less worthy of concern compared to food animals, *d* = −0.36, 95% CI [−0.67, −0.04], *Z* = −2.20, *p* = .028. There were no differences in terms of concern between unappealing wild animals and food animals, *d* = −0.31, 95% CI [−0.73, 0.10], *Z* = −1.49, *p* = .135. Turning to words denoting value, appealing wild animals were no different to food animals, *d* = 0.44, 95% CI [−0.03, 0.91], *Z* = 1.83, *p* = .068, but were represented as more valuable compared to unappealing wild animals, *d* = 0.41, 95% CI [0.06, 0.76], *Z* = 2.29, *p* = .022. We found no differences between food animals and unappealing wild animals in terms of value, *d* = −0.09, 95% CI [−0.40, 0.21], *Z* = −0.60, *p* = .548. Taken together, there were few large or consistent differences with regards to how appealing wild animals, food animals, and unappealing wild animals were represented.

To contextualize the magnitude of the differences between humans and animals, we examined how a set of worthless objects were represented in terms of concern and value: *rock*, *stone*, *rubble*, *debris*, *junk*, *dust*, *rubbish*, *waste*, *trash*, *scrap*, *object*, *garbage*. These scores provide a lower‐bound benchmark that can be used to examine how similar linguistic representations are to those of worthless objects. As expected, humans were represented as substantially more worthy of concern and value compared to these objects, *d* = 1.30, 95% CI [0.44, 2.15], *Z* = 2.98, *p* = .003, *d* = 1.35, 95% CI [0.77, 1.92], *Z* = 4.60, *p* < .001. The magnitude of these differences is about 25–200% larger than the significant differences we observed between humans and other animals.

We explored the relationship between concern and value, finding that WEAT scores reflecting concern (vs. indifference) and value (vs. valueless) were correlated, *r* = .60, 95% CI [.34, .78], *Z* = 3.95, *p* < .001. This is consistent with the idea that concern and value capture aspects of a common construct: moral standing.

Finally, we explored if our effects varied across models, reflecting differences in language derived from informational websites, social media, books, films, and conversation. There was some evidence of heterogeneity in some of the analyses (*I* ^2^s range from 14% to 84%; see Figures S1–S14). When testing for anthropocentric speciesism (Figures S1–S8), some comparisons found significant (*p <* .050) heterogeneity across data sources and models, however, many did not. Likewise, when testing for companion animal speciesism (Figures S9–S14), half of the comparisons found significant heterogeneity (*p <* .050), whilst the other half did not. Overall, these analyses failed to reveal any consistent patterns to suggest that either anthropocentric or companion animal speciesism wnotably stronger, or weaker, across models or mediums. For this reason, we refrain from making any firm statements about whether our conclusions are contingent on a specific model or medium (i.e., informational websites vs. social media vs. books vs. films vs. conversation).

## DISCUSSION

We examined how humans and animals were represented in everyday language by using word embeddings to quantify archives of billions of words derived from the internet, books, films, television, and conversation. We found evidence of anthropocentric speciesism, in that words denoting concern and value were more closely associated with words denoting humans compared to many other animal groups. We also found evidence of what we refer to as companion animal speciesism, in that the same words were more closely associated with words denoting companion animals compared to many other animal groups. We discuss the results in detail below.

### Speciesism is evident in everyday language

The findings largely supported both hypotheses, providing evidence of anthropocentric and companion animal speciesism in everyday language. This is reminiscent of the greater moral concern people express for humans compared to animals (Caviola et al., 2018, 2022; Crimston et al., 2016; Gray et al., 2007; Wilks et al., 2021) and the unique status we afford companion animals (Krings et al., 2021; Leite et al., 2019; Possidónio et al., 2019). The fact that these hypotheses were confirmed across conversation, TV, films, books, and the Internet, leads us to construe speciesism as a ‘collective representation’ (Durkheim, 1972; Moscovici, 1988). As a whole, we take this as strong evidence in support of the idea that speciesism is reflected in language (Dunayer, 2004; Goodall, 1990; PETA, 2021; Singer, 2009a).

The findings are not simply a repackaging of what we already know from self‐reported beliefs (Caviola et al., 2018, 2022; Crimston et al., 2016; Gray et al., 2007; Krings et al., 2021; Leite et al., 2019; Possidónio et al., 2019), moral‐dilemma judgements (Caviola et al., 2020, 2022; Wilks et al., 2021), reaction‐time tasks (Buckels & Trapnell, 2013; Columb & Plant, 2019; Saminaden et al., 2010), and meat‐related dissonance (Bastian et al., 2012; Loughnan et al., 2010; Piazza et al., 2015). By demonstrating the presence and pervasiveness of speciesism in language, the findings speak to the nature of speciesism in a way that prior methods do not. Speciesism can be understood as a socially acquired ideology (Wilks et al., 2021), meaning it is likely to be transmitted, at least in part, via language (Mesoudi et al., 2006). This perspective is important because it provides an explanation for why speciesism is commonplace (Caviola et al., 2018; Dhont et al., 2020) and why children show signs of becoming more speciesist with age (Kozachenko & Piazza, 2022; McGuire et al., 2022; Wilks et al., 2021). Crucially though, it presupposes that speciesism is present and pervasive in language. This assumption demands an analysis of *language*. By providing such an analysis, our work contributes to the literature on speciesism in a novel and important way.

### Caveats, limitations, and generalizability

Although the findings largely confirmed our predictions, there were some noteworthy exceptions. Companion animals were similar to humans in terms of concern but different in terms of value. At the same time, appealing animals were similar to companion animals in terms of value but not concern. These effects could reflect subtly different forms of moral standing (Bastian & Crimston, 2016). The greater concern we see for companion animals may reflect moral standing that flows from social‐embeddedness. Our relationships with companion animals are fundamentally social and involve caring for them (Herzog, 2010; Serpel, 2018) and considering them as part of the family (Voith et al., 1992). The greater value we see for appealing to wild animals, like dolphins, might instead flow from possessing certain properties that are perceived to make the species valuable and worthy of preservation, such as intelligence or beauty (Klebl et al., 2021; Leach et al., 2021).

It is not clear whether our findings reflect patterns of language that shape how people think (Caliskan & Lewis, 2020). There is some indirect evidence to support this idea. Collective linguistic representations, of the sort we identified here, tend to mirror what is captured by conventional measures of mental association (Caliskan et al., 2017). There is also evidence that social learning can be driven by the types of linguistic co‐occurrences that word embedding models capture (Saffran & Kirkham, 2018). Further evidence is required, however, to draw causal conclusions about whether these patterns in language shape how people think about animals.

It seems likely that the results are generalizable. We examined corpora documenting language in informational websites, social media, books, films, and conversation. In some cases, these corpora were as large as 100 billion words (Mikolov et al., 2017; Mikolov, Sutskever, et al., 2013). We also tried to select words representing constructs of interest (humans, animals, concern, etc.) in the most defensible manner we could – guided by prior work (Caliskan et al., 2017; Charlesworth et al., 2021; Leite et al., 2019) and models of language (Miller, 1995). The breadth and depth of the analysis suggest that the results reflect prevailing linguistic representations of humans and animals. That said, future work could examine larger sets of validated animal words and if similar trends are evident in languages other than English.

We found evidence of speciesism in the co‐occurrence relationships of words denoting concern and value and those denoting humans and other animals. There is good reason to believe that these statistics are meaningful abstractions that reflect collective expressions of human psychology (Caliskan et al., 2017; Caliskan & Lewis, 2020; Charlesworth et al., 2021; Garg et al., 2018; Lewis & Lupyan, 2020). An analogous approach could be used to test claims about the role that language plays in the treatment of marginalized human groups by examining if they are derogated in language in a similar way as are animals. Indeed, many forms of prejudice are thought to be underpinned by biased patterns of language use (Haslam et al., 2016, 2020; Maass et al., 2014; Sutton, 2010) that may ultimately be rooted in the same processes as speciesism (Dhont et al., 2014, 2016; Jackson, 2019). This approach may also open up the possibility of examining how language has changed over time by delving into historical archives (Garg et al., 2018).

It is important to note that the linguistic co‐occurrences we analysed are but one way that speciesism might manifest in language. Other perspectives suggest that speciesism is found in grammar and metaphor; for example in the collective tendency to refer to animals as ‘it’ as opposed to ‘he’ or ‘she’ (Dunayer, 2004; Goodall, 1990; PETA, 2021; Sealey & Oakley, 2013; Singer, 2009a). Moreover, it could be interesting to explore if language encodes other disadvantageous distinctions which may ground animals' moral standing, like their intelligence and competence (Gray et al., 2007; Leach et al., 2021; Sevillano & Fiske, 2016).

## CONCLUSION

We analysed how humans and animals were represented in everyday language by applying machine learning techniques to billions of words derived from various social products. These analyses revealed that words denoting concern (vs. indifference) and value (vs. valueless) were more closely associated with words denoting humans compared to most other animal groups; and companion animals compared to most other animal groups. The findings suggest that speciesism can be construed as a collective representation detectable in the language of conversation, TV, film, books, social media, news reporting, and informational websites.

## AUTHOR CONTRIBUTIONS

**Stefan Leach:** Conceptualization; data curation; formal analysis; investigation; methodology; project administration; resources; software; validation; visualization; writing – original draft; writing – review and editing. **Andrew P. Kitchin:** Data curation; formal analysis; investigation; methodology; software; validation; writing – review and editing. **Robbie M. Sutton:** Funding acquisition; writing – review and editing. **Kristof Dhont:** Funding acquisition; writing – review and editing.

## CONFLICTS OF INTEREST

The authors declare no conflict of interest.

### OPEN RESEARCH BADGES

This article has earned Open Data and Open Materials badges. Data and materials are available at https://osf.io/nzsr3/.

## Supporting information


Appendix S1

## Data Availability

The methods, data, and analysis script are available via the *Open Science Framework* (https://osf.io/nzsr3/).

## Footnotes

^1^
Because off‐the‐shelf word lists denoting positivity and negativity were readily available (Caliskan et al., 2017; Charlesworth et al., 2021), we also analysed how humans and animals were represented along this dimension. Descriptively, the pattern of results reflected a graded continuum, with humans represented in the most positive terms, followed by companion animals, appealing wild animals, food animals, and finally unappealing wild animals‐‐although not all groups differed significantly from one another. See the Supplemental File for further details.
