# Supplementary material for: Speciesism in everyday language
Source: Br J Soc Psychol. 2022 Jul 30;62(1):486–502. doi: 10.1111/bjso.12561 (PMC10086848; doi:10.1111/bjso.12561)
Supplement: Supplementary file 1 — Appendix S1 [file BJSO-62-486-s001.docx]

**Speciesism in everyday language**

**Supplemental File**

**Table S1**

Available vectors.

|  | Internet (General) | Internet (News) | Internet (Wikipedia + News) | Internet (Twitter) | TV and Film | Books | Speech |
| --- | --- | --- | --- | --- | --- | --- | --- |
| care | *✓* | *✓* | *✓* | *✓* | *✓* | *✓* | *✓* |
| cares | *X* | *✓* | *✓* | *✓* | *✓* | *X* | *✓* |
| caring | *✓* | *✓* | *✓* | *✓* | *✓* | *✓* | *✓* |
| cared | *✓* | *✓* | *✓* | *✓* | *✓* | *✓* | *✓* |
| concern | *✓* | *✓* | *✓* | *✓* | *✓* | *✓* | *✓* |
| concerns | *X* | *✓* | *✓* | *✓* | *✓* | *X* | *X* |
| concerned | *✓* | *✓* | *✓* | *✓* | *✓* | *✓* | *✓* |
| concerning | *✓* | *✓* | *✓* | *✓* | *✓* | *✓* | *✓* |
| aid | *✓* | *✓* | *✓* | *✓* | *✓* | *✓* | *✓* |
| aids | *✓* | *✓* | *✓* | *✓* | *✓* | *X* | *✓* |
| aided | *✓* | *✓* | *✓* | *✓* | *✓* | *X* | *✓* |
| aiding | *✓* | *✓* | *✓* | *✓* | *✓* | *X* | *✓* |
| tend | *✓* | *✓* | *✓* | *✓* | *✓* | *✓* | *✓* |
| tends | *✓* | *✓* | *✓* | *✓* | *✓* | *✓* | *✓* |
| tending | *✓* | *✓* | *✓* | *✓* | *✓* | *✓* | *✓* |
| tended | *✓* | *✓* | *✓* | *✓* | *✓* | *✓* | *✓* |
| help | *✓* | *✓* | *✓* | *✓* | *✓* | *✓* | *✓* |
| helps | *X* | *✓* | *✓* | *✓* | *✓* | *X* | *✓* |
| helping | *✓* | *✓* | *✓* | *✓* | *✓* | *✓* | *✓* |
| helped | *✓* | *✓* | *✓* | *✓* | *✓* | *✓* | *✓* |
| assist | *✓* | *✓* | *✓* | *✓* | *✓* | *✓* | *✓* |
| assists | *X* | *✓* | *✓* | *✓* | *✓* | *X* | *X* |
| assisting | *✓* | *✓* | *✓* | *✓* | *✓* | *X* | *✓* |
| assisted | *✓* | *✓* | *✓* | *✓* | *✓* | *X* | *✓* |
| sympathy | *✓* | *✓* | *✓* | *✓* | *✓* | *✓* | *✓* |
| sympathize | *✓* | *✓* | *✓* | *✓* | *✓* | *X* | *✓* |
| sympathizes | *✓* | *✓* | *✓* | *✓* | *✓* | *X* | *X* |
| sympathized | *✓* | *✓* | *✓* | *X* | *✓* | *X* | *X* |
| sympathetic | *✓* | *✓* | *✓* | *✓* | *✓* | *✓* | *✓* |
| sympathetically | *✓* | *✓* | *✓* | *✓* | *✓* | *X* | *✓* |
| compassion | *✓* | *✓* | *✓* | *✓* | *✓* | *✓* | *✓* |
| compassions | *X* | *✓* | *✓* | *✓* | *X* | *X* | *X* |
| compassionate | *✓* | *✓* | *✓* | *✓* | *✓* | *X* | *✓* |
| apathy | *✓* | *✓* | *✓* | *✓* | *✓* | *✓* | *X* |
| apathetic | *✓* | *✓* | *✓* | *✓* | *✓* | *X* | *X* |
| uncaring | *X* | *✓* | *✓* | *✓* | *✓* | *X* | *✓* |
| unaffectionate | *X* | *✓* | *✓* | *X* | *X* | *X* | *X* |
| indifference | *✓* | *✓* | *✓* | *✓* | *✓* | *X* | *✓* |
| indifferent | *✓* | *✓* | *✓* | *✓* | *✓* | *✓* | *✓* |
| unconcern | *✓* | *✓* | *✓* | *X* | *✓* | *X* | *X* |
| unconcerned | *✓* | *✓* | *✓* | *✓* | *✓* | *X* | *✓* |
| disregard | *✓* | *✓* | *✓* | *✓* | *✓* | *✓* | *✓* |
| disregards | *X* | *✓* | *✓* | *✓* | *✓* | *X* | *X* |
| disregarded | *✓* | *✓* | *✓* | *✓* | *✓* | *X* | *X* |
| disregarding | *✓* | *✓* | *✓* | *✓* | *✓* | *X* | *X* |
| detach | *✓* | *✓* | *✓* | *✓* | *✓* | *X* | *✓* |
| detaches | *✓* | *✓* | *✓* | *✓* | *✓* | *X* | *✓* |
| detaching | *✓* | *✓* | *✓* | *✓* | *✓* | *X* | *X* |
| detached | *✓* | *✓* | *✓* | *✓* | *✓* | *X* | *✓* |
| neglect | *✓* | *✓* | *✓* | *✓* | *✓* | *✓* | *✓* |
| neglects | *X* | *✓* | *✓* | *✓* | *✓* | *X* | *X* |
| neglected | *✓* | *✓* | *✓* | *✓* | *✓* | *✓* | *✓* |
| neglecting | *✓* | *✓* | *✓* | *✓* | *✓* | *X* | *X* |
| neglectful | *✓* | *✓* | *✓* | *✓* | *✓* | *X* | *X* |
| value | *✓* | *✓* | *✓* | *✓* | *✓* | *✓* | *✓* |
| valuable | *✓* | *✓* | *✓* | *✓* | *✓* | *✓* | *✓* |
| valued | *✓* | *✓* | *✓* | *✓* | *✓* | *✓* | *✓* |
| valuing | *✓* | *✓* | *✓* | *✓* | *✓* | *X* | *X* |
| values | *X* | *✓* | *✓* | *✓* | *✓* | *X* | *X* |
| appreciate | *✓* | *✓* | *✓* | *✓* | *✓* | *✓* | *✓* |
| appreciates | *✓* | *✓* | *✓* | *✓* | *✓* | *X* | *✓* |
| appreciated | *✓* | *✓* | *✓* | *✓* | *✓* | *✓* | *✓* |
| appreciating | *✓* | *✓* | *✓* | *✓* | *✓* | *X* | *✓* |
| precious | *✓* | *✓* | *✓* | *✓* | *✓* | *✓* | *✓* |
| preciously | *✓* | *✓* | *✓* | *X* | *✓* | *X* | *X* |
| priceless | *✓* | *✓* | *✓* | *✓* | *✓* | *X* | *✓* |
| invaluable | *✓* | *✓* | *✓* | *✓* | *✓* | *X* | *✓* |
| important | *✓* | *✓* | *✓* | *✓* | *✓* | *✓* | *✓* |
| importance | *✓* | *✓* | *✓* | *✓* | *✓* | *✓* | *✓* |
| importantly | *✓* | *✓* | *✓* | *✓* | *✓* | *✓* | *✓* |
| worth | *✓* | *✓* | *✓* | *✓* | *✓* | *✓* | *✓* |
| worthy | *✓* | *✓* | *✓* | *✓* | *✓* | *✓* | *✓* |
| worthiness | *✓* | *✓* | *✓* | *✓* | *✓* | *X* | *✓* |
| significant | *✓* | *✓* | *✓* | *✓* | *✓* | *✓* | *✓* |
| significantly | *✓* | *✓* | *✓* | *✓* | *✓* | *✓* | *✓* |
| significance | *✓* | *✓* | *✓* | *✓* | *✓* | *✓* | *✓* |
| cherish | *✓* | *✓* | *✓* | *✓* | *✓* | *X* | *✓* |
| cherished | *✓* | *✓* | *✓* | *✓* | *✓* | *X* | *✓* |
| cherishes | *✓* | *✓* | *✓* | *✓* | *✓* | *X* | *X* |
| cherishing | *✓* | *✓* | *✓* | *✓* | *✓* | *X* | *X* |
| valueless | *✓* | *✓* | *✓* | *✓* | *✓* | *X* | *X* |
| worthless | *✓* | *✓* | *✓* | *✓* | *✓* | *✓* | *✓* |
| worthlessness | *✓* | *✓* | *✓* | *✓* | *✓* | *X* | *X* |
| insignificant | *✓* | *✓* | *✓* | *✓* | *✓* | *X* | *✓* |
| insignificantly | *X* | *✓* | *✓* | *X* | *✓* | *X* | *X* |
| meritless | *X* | *✓* | *✓* | *X* | *✓* | *X* | *X* |
| unimportant | *✓* | *✓* | *✓* | *✓* | *✓* | *X* | *✓* |
| unimportance | *✓* | *✓* | *✓* | *X* | *✓* | *X* | *X* |
| unimportantly | *X* | *✓* | *X* | *X* | *X* | *X* | *X* |
| deficient | *✓* | *✓* | *✓* | *✓* | *✓* | *✓* | *X* |
| deficiency | *✓* | *✓* | *✓* | *✓* | *✓* | *X* | *✓* |
| insufficient | *✓* | *✓* | *✓* | *✓* | *✓* | *X* | *✓* |
| inferior | *✓* | *✓* | *✓* | *✓* | *✓* | *✓* | *✓* |
| substandard | *X* | *✓* | *✓* | *✓* | *✓* | *X* | *X* |
| lack | *✓* | *✓* | *✓* | *✓* | *✓* | *✓* | *✓* |
| lacks | *X* | *✓* | *✓* | *✓* | *✓* | *X* | *X* |
| lacked | *✓* | *✓* | *✓* | *✓* | *✓* | *X* | *✓* |
| lacking | *✓* | *✓* | *✓* | *✓* | *✓* | *✓* | *✓* |
| disfavour | *✓* | *✓* | *✓* | *X* | *✓* | *X* | *X* |
| disfavours | *X* | *✓* | *✓* | *X* | *X* | *X* | *X* |
| disfavored | *X* | *✓* | *✓* | *X* | *✓* | *X* | *X* |
| disfavouring | *X* | *X* | *✓* | *X* | *X* | *X* | *X* |
| useless | *✓* | *✓* | *✓* | *✓* | *✓* | *✓* | *✓* |
| uselessness | *✓* | *✓* | *✓* | *✓* | *✓* | *X* | *X* |
| inutility | *✓* | *✓* | *✓* | *X* | *X* | *X* | *X* |
| caress | *✓* | *✓* | *✓* | *✓* | *✓* | *X* | *✓* |
| freedom | *✓* | *✓* | *✓* | *✓* | *✓* | *✓* | *✓* |
| health | *✓* | *✓* | *✓* | *✓* | *✓* | *✓* | *✓* |
| love | *✓* | *✓* | *✓* | *✓* | *✓* | *✓* | *✓* |
| peace | *✓* | *✓* | *✓* | *✓* | *✓* | *✓* | *✓* |
| cheer | *✓* | *✓* | *✓* | *✓* | *✓* | *✓* | *✓* |
| friend | *✓* | *✓* | *✓* | *✓* | *✓* | *✓* | *✓* |
| heaven | *✓* | *✓* | *✓* | *✓* | *✓* | *✓* | *✓* |
| loyal | *✓* | *✓* | *✓* | *✓* | *✓* | *✓* | *✓* |
| pleasure | *✓* | *✓* | *✓* | *✓* | *✓* | *✓* | *✓* |
| diamond | *✓* | *✓* | *✓* | *✓* | *✓* | *✓* | *✓* |
| gentle | *✓* | *✓* | *✓* | *✓* | *✓* | *✓* | *✓* |
| honest | *✓* | *✓* | *✓* | *✓* | *✓* | *✓* | *✓* |
| lucky | *✓* | *✓* | *✓* | *✓* | *✓* | *✓* | *✓* |
| rainbow | *✓* | *✓* | *✓* | *✓* | *✓* | *✓* | *✓* |
| diploma | *✓* | *✓* | *✓* | *✓* | *✓* | *✓* | *✓* |
| gift | *✓* | *✓* | *✓* | *✓* | *✓* | *✓* | *✓* |
| honor | *✓* | *✓* | *✓* | *✓* | *✓* | *✓* | *✓* |
| miracle | *✓* | *✓* | *✓* | *✓* | *✓* | *✓* | *✓* |
| sunrise | *✓* | *✓* | *✓* | *✓* | *✓* | *✓* | *✓* |
| family | *✓* | *✓* | *✓* | *✓* | *✓* | *✓* | *✓* |
| happy | *✓* | *✓* | *✓* | *✓* | *✓* | *✓* | *✓* |
| happiness | *✓* | *✓* | *✓* | *✓* | *✓* | *✓* | *✓* |
| laughter | *✓* | *✓* | *✓* | *✓* | *✓* | *✓* | *✓* |
| paradise | *✓* | *✓* | *✓* | *✓* | *✓* | *✓* | *✓* |
| vacation | *✓* | *✓* | *✓* | *✓* | *✓* | *✓* | *✓* |
| fun | *✓* | *✓* | *✓* | *✓* | *✓* | *✓* | *✓* |
| fantastic | *✓* | *✓* | *✓* | *✓* | *✓* | *✓* | *✓* |
| lovable | *✓* | *✓* | *✓* | *✓* | *✓* | *✓* | *✓* |
| magical | *✓* | *✓* | *✓* | *✓* | *✓* | *✓* | *✓* |
| delight | *✓* | *✓* | *✓* | *✓* | *✓* | *✓* | *✓* |
| joy | *✓* | *✓* | *✓* | *✓* | *✓* | *✓* | *✓* |
| relaxing | *✓* | *✓* | *✓* | *✓* | *✓* | *✓* | *✓* |
| excited | *✓* | *✓* | *✓* | *✓* | *✓* | *✓* | *✓* |
| lover | *✓* | *✓* | *✓* | *✓* | *✓* | *✓* | *✓* |
| cheerful | *✓* | *✓* | *✓* | *✓* | *✓* | *✓* | *✓* |
| abuse | *✓* | *✓* | *✓* | *✓* | *✓* | *✓* | *✓* |
| crash | *✓* | *✓* | *✓* | *✓* | *✓* | *✓* | *✓* |
| filth | *✓* | *✓* | *✓* | *✓* | *✓* | *X* | *✓* |
| murder | *✓* | *✓* | *✓* | *✓* | *✓* | *✓* | *✓* |
| sickness | *✓* | *✓* | *✓* | *✓* | *✓* | *✓* | *✓* |
| accident | *✓* | *✓* | *✓* | *✓* | *✓* | *✓* | *✓* |
| death | *✓* | *✓* | *✓* | *✓* | *✓* | *✓* | *✓* |
| grief | *✓* | *✓* | *✓* | *✓* | *✓* | *✓* | *✓* |
| poison | *✓* | *✓* | *✓* | *✓* | *✓* | *✓* | *✓* |
| stink | *✓* | *✓* | *✓* | *✓* | *✓* | *✓* | *✓* |
| assault | *✓* | *✓* | *✓* | *✓* | *✓* | *✓* | *✓* |
| disaster | *✓* | *✓* | *✓* | *✓* | *✓* | *✓* | *✓* |
| hatred | *✓* | *✓* | *✓* | *✓* | *✓* | *✓* | *✓* |
| pollute | *✓* | *✓* | *✓* | *✓* | *✓* | *✓* | *✓* |
| tragedy | *✓* | *✓* | *✓* | *✓* | *✓* | *✓* | *✓* |
| divorce | *✓* | *✓* | *✓* | *✓* | *✓* | *✓* | *✓* |
| jail | *✓* | *✓* | *✓* | *✓* | *✓* | *✓* | *✓* |
| poverty | *✓* | *✓* | *✓* | *✓* | *✓* | *✓* | *✓* |
| ugly | *✓* | *✓* | *✓* | *✓* | *✓* | *✓* | *✓* |
| cancer | *✓* | *✓* | *✓* | *✓* | *✓* | *✓* | *✓* |
| kill | *✓* | *✓* | *✓* | *✓* | *✓* | *✓* | *✓* |
| rotten | *✓* | *✓* | *✓* | *✓* | *✓* | *✓* | *✓* |
| vomit | *✓* | *✓* | *✓* | *✓* | *✓* | *✓* | *✓* |
| agony | *✓* | *✓* | *✓* | *✓* | *✓* | *X* | *✓* |
| prison | *✓* | *✓* | *✓* | *✓* | *✓* | *✓* | *✓* |
| torture | *✓* | *✓* | *✓* | *✓* | *✓* | *✓* | *✓* |
| wreck | *✓* | *✓* | *✓* | *✓* | *✓* | *✓* | *✓* |
| die | *✓* | *✓* | *✓* | *✓* | *✓* | *✓* | *✓* |
| disease | *✓* | *✓* | *✓* | *✓* | *✓* | *✓* | *✓* |
| mourning | *✓* | *✓* | *✓* | *✓* | *✓* | *✓* | *✓* |
| virus | *✓* | *✓* | *✓* | *✓* | *✓* | *✓* | *✓* |
| killer | *✓* | *✓* | *✓* | *✓* | *✓* | *✓* | *✓* |
| nightmare | *✓* | *✓* | *✓* | *✓* | *✓* | *✓* | *✓* |
| stress | *✓* | *✓* | *✓* | *✓* | *✓* | *✓* | *✓* |

**Figure S1**

Model- and meta-level estimates for WEAT (concern - indifference) comparing humans with companion animals.


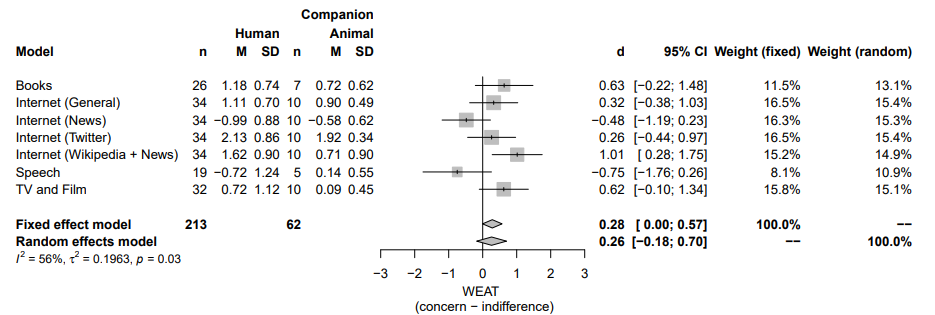


*Note.* Figure depicts means (points), model-level 95% CIs (whiskers), weights (shaded boxes), and meta-level estimates (diamonds). Model is fit via the *meta* (Schwarzer, 2007) package for R (R Core Team, 2020). Inverse variance method is used for pooling.

**Figure S2**

Model- and meta-level estimates for WEAT (concern - indifference) comparing humans with appealing wild animals.


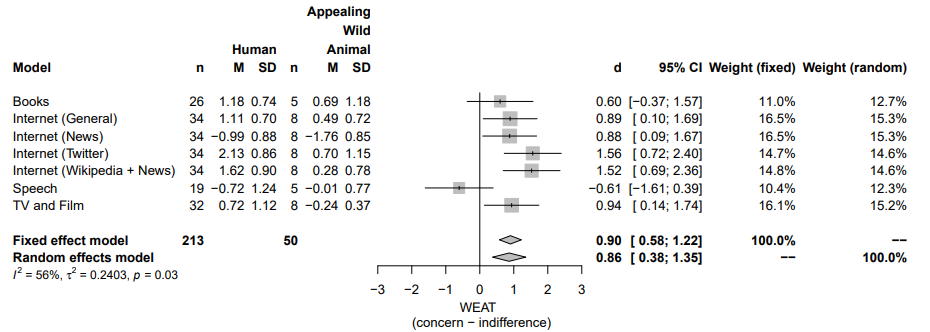


*Note.* Figure depicts means (points), model-level 95% CIs (whiskers), weights (shaded boxes), and meta-level estimates (diamonds). Model is fit via the *meta* (Schwarzer, 2007) package for R (R Core Team, 2020). Inverse variance method is used for pooling.

**Figure S3**

Model- and meta-level estimates for WEAT (concern - indifference) comparing humans with food animals.


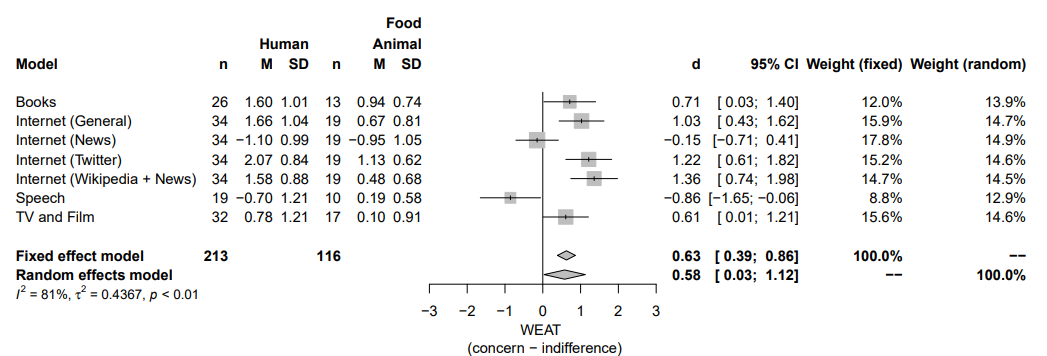


*Note.* Figure depicts means (points), model-level 95% CIs (whiskers), weights (shaded boxes), and meta-level estimates (diamonds). Model is fit via the *meta* (Schwarzer, 2007) package for R (R Core Team, 2020). Inverse variance method is used for pooling.

**Figure S4**

Model- and meta-level estimates for WEAT (concern - indifference) comparing humans with unappealing wild animals.


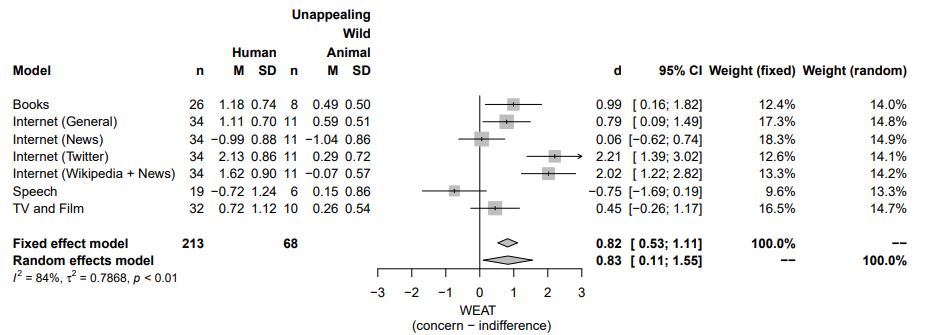


*Note.* Figure depicts means (points), model-level 95% CIs (whiskers), weights (shaded boxes), and meta-level estimates (diamonds). Model is fit via the *meta* (Schwarzer, 2007) package for R (R Core Team, 2020). Inverse variance method is used for pooling.

**Figure S5**

Model- and meta-level estimates for WEAT (value - valueless) comparing humans with companion animals.


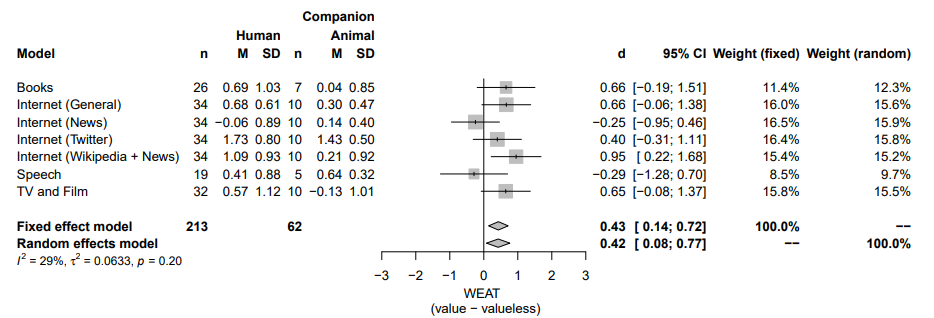


*Note.* Figure depicts means (points), model-level 95% CIs (whiskers), weights (shaded boxes), and meta-level estimates (diamonds). Model is fit via the *meta* (Schwarzer, 2007) package for R (R Core Team, 2020). Inverse variance method is used for pooling.

**Figure S6**

Model- and meta-level estimates for WEAT (value - valueless) comparing humans with appealing wild animals.


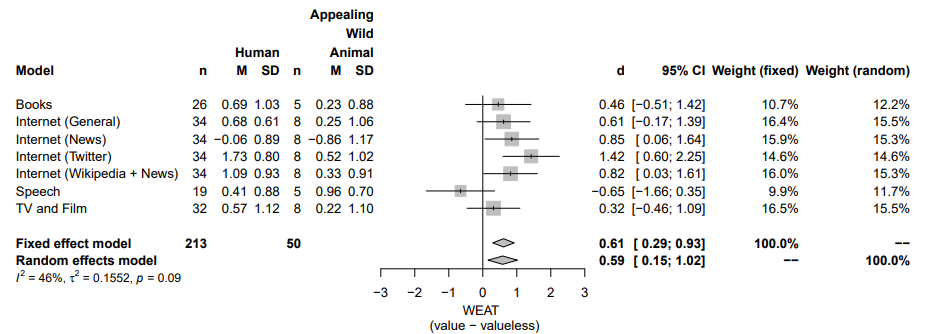


*Note.* Figure depicts means (points), model-level 95% CIs (whiskers), weights (shaded boxes), and meta-level estimates (diamonds). Model is fit via the *meta* (Schwarzer, 2007) package for R (R Core Team, 2020). Inverse variance method is used for pooling.

**Figure S7**

Model- and meta-level estimates for WEAT (value - valueless) comparing humans with food animals.


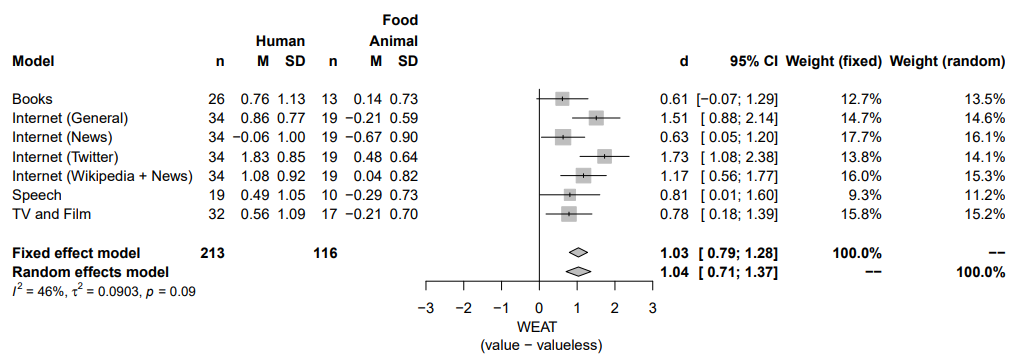


*Note.* Figure depicts means (points), model-level 95% CIs (whiskers), weights (shaded boxes), and meta-level estimates (diamonds). Model is fit via the *meta* (Schwarzer, 2007) package for R (R Core Team, 2020). Inverse variance method is used for pooling.

**Figure S8**

Model- and meta-level estimates for WEAT (value - valueless) comparing humans with unappealing wild animals.


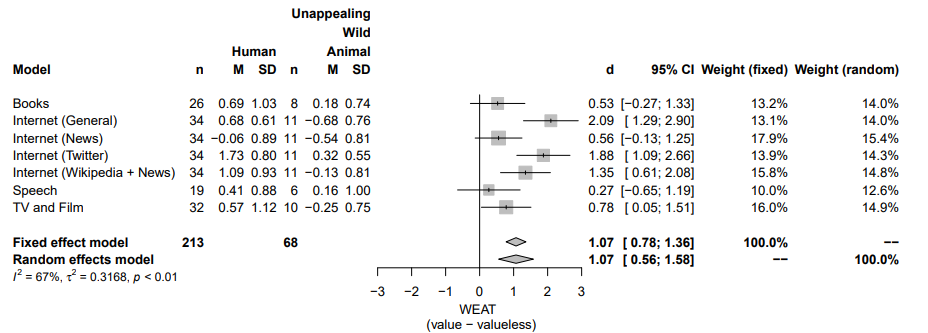


*Note.* Figure depicts means (points), model-level 95% CIs (whiskers), weights (shaded boxes), and meta-level estimates (diamonds). Model is fit via the *meta* (Schwarzer, 2007) package for R (R Core Team, 2020). Inverse variance method is used for pooling.

**Figure S9**

Model- and meta-level estimates for WEAT (concern - indifference) comparing companion animals with appealing wild animals.


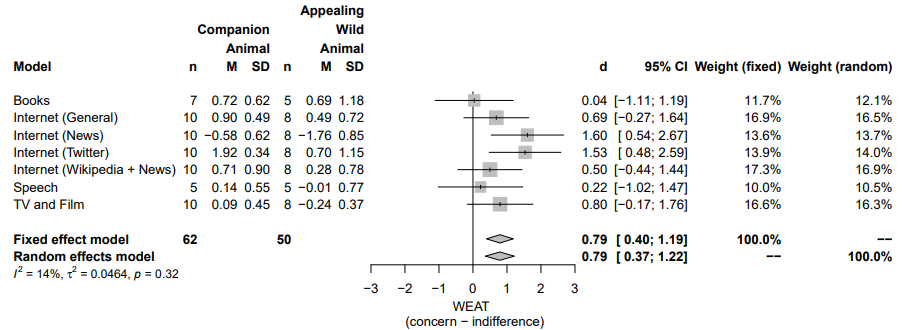


*Note.* Figure depicts means (points), model-level 95% CIs (whiskers), weights (shaded boxes), and meta-level estimates (diamonds). Model is fit via the *meta* (Schwarzer, 2007) package for R (R Core Team, 2020). Inverse variance method is used for pooling.

**Figure S10**

Model- and meta-level estimates for WEAT (concern - indifference) comparing companion animals with food animals.


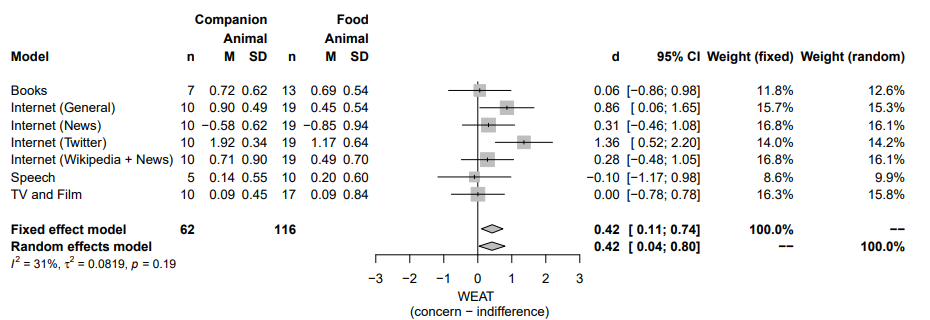


*Note.* Figure depicts means (points), model-level 95% CIs (whiskers), weights (shaded boxes), and meta-level estimates (diamonds). Model is fit via the *meta* (Schwarzer, 2007) package for R (R Core Team, 2020). Inverse variance method is used for pooling.

**Figure S11**

Model- and meta-level estimates for WEAT (concern - indifference) comparing companion animals with unappealing wild animals.


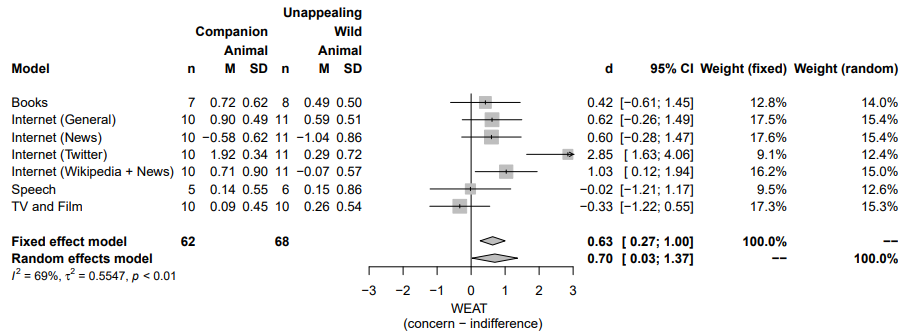


*Note.* Figure depicts means (points), model-level 95% CIs (whiskers), weights (shaded boxes), and meta-level estimates (diamonds). Model is fit via the *meta* (Schwarzer, 2007) package for R (R Core Team, 2020). Inverse variance method is used for pooling.

**Figure S12**

Model- and meta-level estimates for WEAT (value - valueless) comparing companion animals with appealing wild animals.


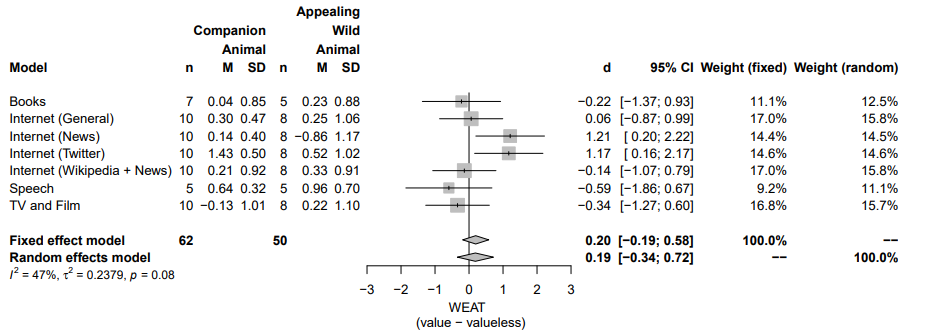


*Note.* Figure depicts means (points), model-level 95% CIs (whiskers), weights (shaded boxes), and meta-level estimates (diamonds). Model is fit via the *meta* (Schwarzer, 2007) package for R (R Core Team, 2020). Inverse variance method is used for pooling.

**Figure S13**

Model- and meta-level estimates for WEAT (value - valueless) comparing companion animals with food animals.


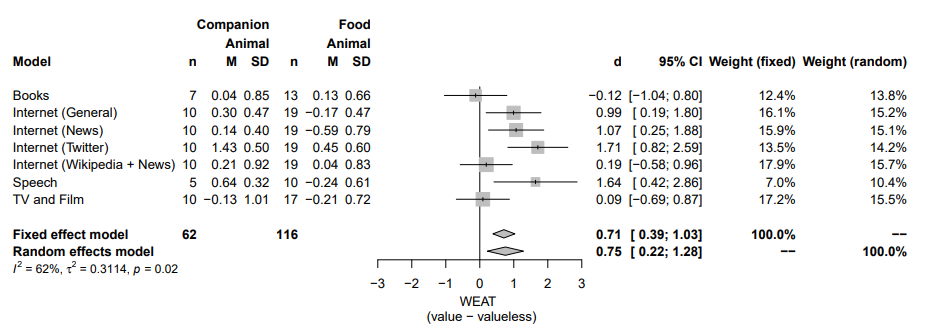


*Note.* Figure depicts means (points), model-level 95% CIs (whiskers), weights (shaded boxes), and meta-level estimates (diamonds). Model is fit via the *meta* (Schwarzer, 2007) package for R (R Core Team, 2020). Inverse variance method is used for pooling.

**Figure S14**

Model- and meta-level estimates for WEAT (value - valueless) comparing companion animals with unappealing wild animals.


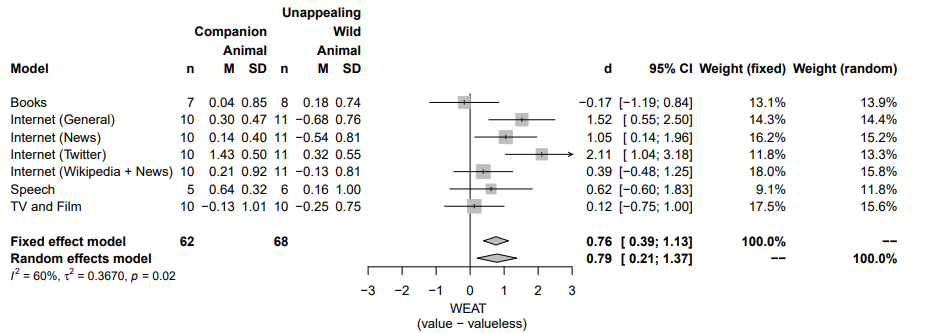


*Note.* Figure depicts means (points), model-level 95% CIs (whiskers), weights (shaded boxes), and meta-level estimates (diamonds). Model is fit via the *meta* (Schwarzer, 2007) package for R (R Core Team, 2020). Inverse variance method is used for pooling.

**Valence**

We explored how humans and animals were represented in terms of their valence. We did this because valence is a broad evaluative dimension that is linked, albeit distally, to perceptions of animals’ moral standing (e.g., Possidónio et al., 2019) and because off-the-shelf word lists denoting positivity and negativity were readily available (Caliskan et al., 2017; Charlesworth et al., 2021). We followed the same WEAT method outlined in the Main Body to explore how humans and animals were represented in terms of their valence. We took words denoting positivity and negativity from prior work (Caliskan et al., 2017; Charlesworth et al., 2021). This work utilised many of the same words to denote positivity and negativity as does work on the Implicit Association Test--an established measure of mental association that often relies on word-based categorization judgements (Greenwald et al., 1998). These words were originally selected on the basis of self-reported pleasantness (Bellezza et al, 1986) and have been shown to perform in the expected manner in tasks like the IAT (Greenwald et al., 1998). The lists are presented in Table S2.

**Table S2**

Words denoting valence.

| Category | Words |
| --- | --- |
| Positivity | caress, freedom, health, love, peace, cheer, friend, heaven, loyal, pleasure, diamond, gentle, honest, lucky, rainbow, diploma, gift, honor, miracle, sunrise, family, happy, happiness, laughter, paradise, vacation, fun, fantastic, lovable, magical, delight, joy, relaxing, excited, lover, cheerful |
| Negativity | abuse, crash, filth, murder, sickness, accident, death, grief, poison, stink, assault, disaster, hatred, pollute, tragedy, divorce, jail, poverty, ugly, cancer, kill, rotten, vomit, agony, prison, torture, wreck, die, disease, disaster, mourning, virus, killer, nightmare, stress |

There was some indication of differences between humans and animals along the dimension of valence. As can be seen in Figure S15, humans were represented as more positive compared to food animals, *d* = 0.48, 95% CI [-0.07, 1.02], Z = 1.72, *p* = .086, and unappealing wild animals, *d* = 0.67, 95% CI [0.14, 1.19], Z = 2.49, *p* = .013. Although there were no differences between humans and companion animals, *d* = -0.01, 95% CI [-0.41, 0.40], Z = -0.04, *p* = .970, nor between humans and appealing wild animals, *d* = 0.22, 95% CI [-0.29, 0.74], Z = 0.85, *p* = .396. Further details can be found in Figure S17-S20.

**Figure S15**

Differences in positivity (vs. negativity) between humans and other animals.


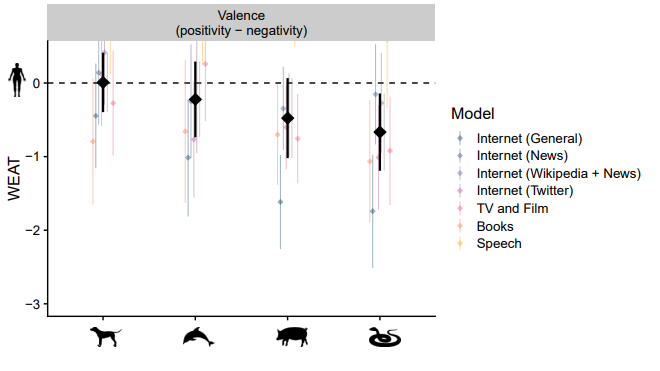


*Note.* WEAT is a standardized measure reflecting the average difference in the similarity between words representing humans and other groups; where y = 0 reflects no difference with humans. The figure depicts model-level estimates (colored diamonds), meta-level estimates (black diamonds), and 95% CIs (whiskers).

There was also evidence of differences between companion-animals and other animals. As can be seen in Figure S16, Companion-animals were represented in more positive terms compared to food animals, *d* = 0.80, 95% CI [0.28, 1.32], Z = 3.02, *p* = .003, and unappealing wild animals, *d* = 1.19, 95% CI [0.70, 1.68], Z = 4.76, *p* < .001. We found no differences between companion animals and appealing wild animals, *d* = 0.52, 95% CI [-0.18, 1.21], Z = 1.46, *p* = .145. Further details can be found in Figure S21-S23.

**Figure S16**

Differences in positivity (vs. negativity) between companion animals and other animals.


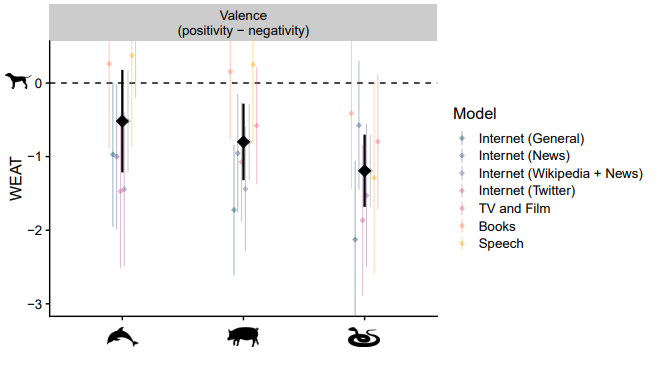


*Note.* WEAT is a standardized measure reflecting the average difference in the similarity between words representing humans and other groups; where y = 0 reflects no difference with companion animals. The figure depicts model-level estimates (colored diamonds), meta-level estimates (black diamonds), and 95% CIs (whiskers).

**Figure S17**

Model- and meta-level estimates for WEAT (positivity - negativity) comparing humans with companion animals.


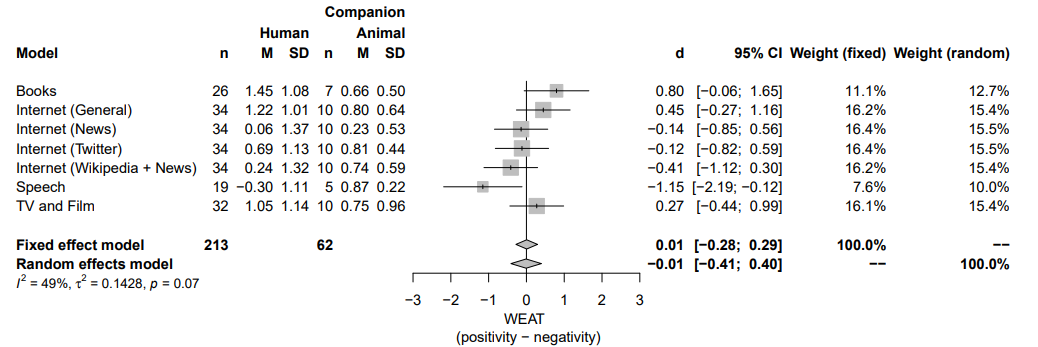


*Note.* Figure depicts means (points), model-level 95% CIs (whiskers), weights (shaded boxes), and meta-level estimates (diamonds). Model is fit via the *meta* (Schwarzer, 2007) package for R (R Core Team, 2020). Inverse variance method is used for pooling.

**Figure S18**

Model- and meta-level estimates for WEAT (positivity - negativity) comparing humans with appealing wild animals.


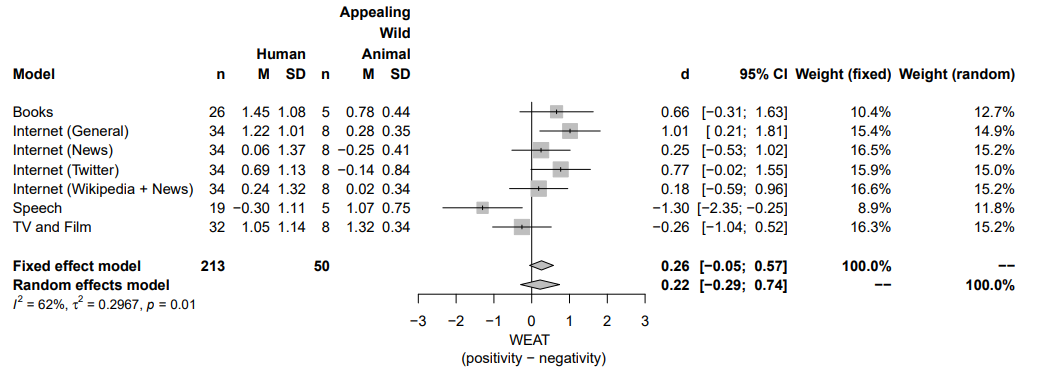


*Note.* Figure depicts means (points), model-level 95% CIs (whiskers), weights (shaded boxes), and meta-level estimates (diamonds). Model is fit via the *meta* (Schwarzer, 2007) package for R (R Core Team, 2020). Inverse variance method is used for pooling.

**Figure S19**

Model- and meta-level estimates for WEAT (positivity - negativity) comparing humans with food animals.


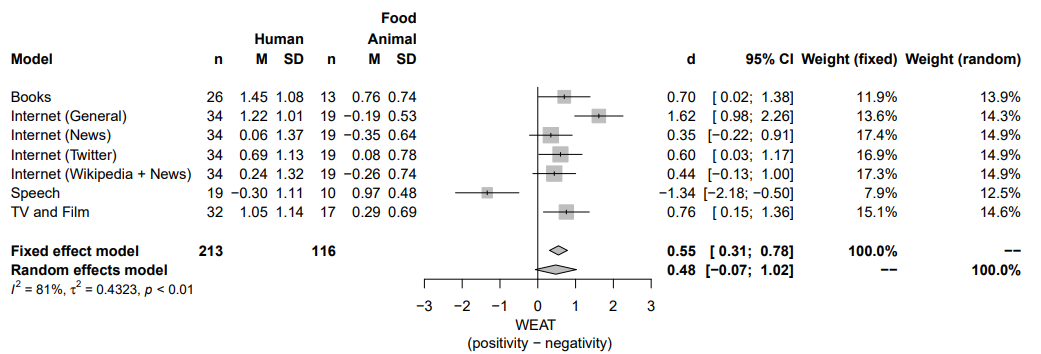


*Note.* Figure depicts means (points), model-level 95% CIs (whiskers), weights (shaded boxes), and meta-level estimates (diamonds). Model is fit via the *meta* (Schwarzer, 2007) package for R (R Core Team, 2020). Inverse variance method is used for pooling.

**Figure S20**

Model- and meta-level estimates for WEAT (positivity - negativity) comparing humans with unappealing wild animals.


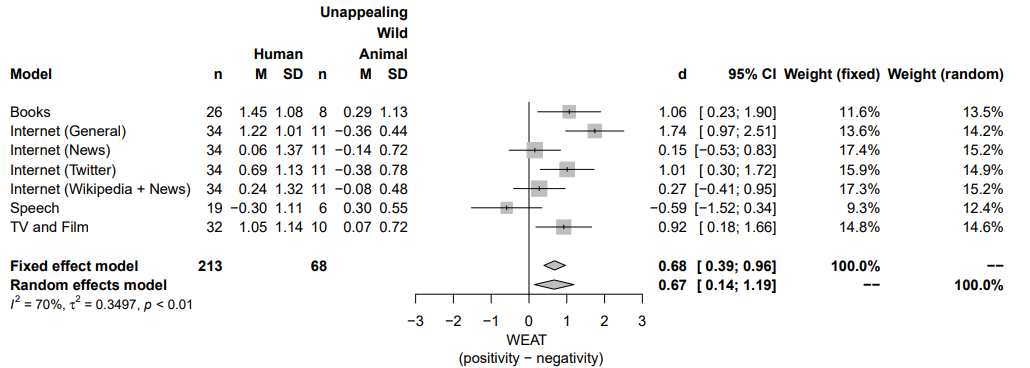


*Note.* Figure depicts means (points), model-level 95% CIs (whiskers), weights (shaded boxes), and meta-level estimates (diamonds). Model is fit via the *meta* (Schwarzer, 2007) package for R (R Core Team, 2020). Inverse variance method is used for pooling.

**Figure S21**

Model- and meta-level estimates for WEAT (positivity - negativity) comparing companion animals with appealing wild animals.


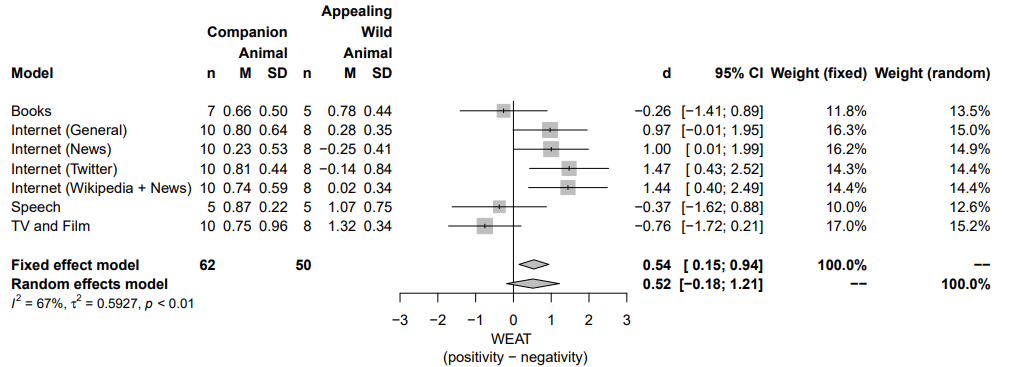


*Note.* Figure depicts means (points), model-level 95% CIs (whiskers), weights (shaded boxes), and meta-level estimates (diamonds). Model is fit via the *meta* (Schwarzer, 2007) package for R (R Core Team, 2020). Inverse variance method is used for pooling.

**Figure S22**

Model- and meta-level estimates for WEAT (positivity - negativity) comparing companion animals with food animals.


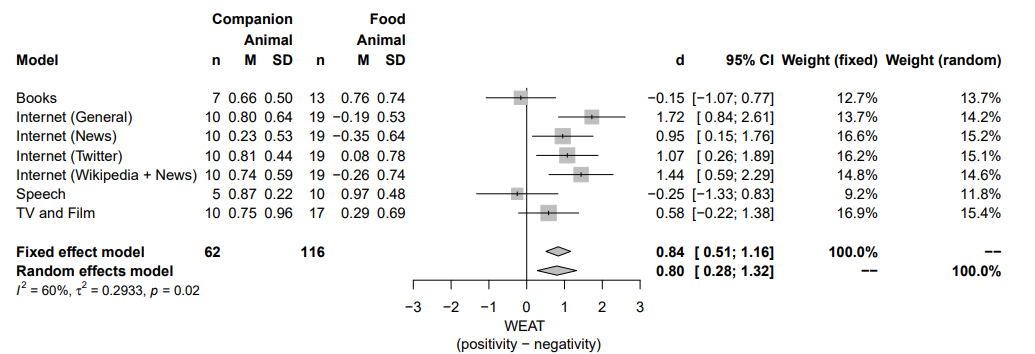


*Note.* Figure depicts means (points), model-level 95% CIs (whiskers), weights (shaded boxes), and meta-level estimates (diamonds). Model is fit via the *meta* (Schwarzer, 2007) package for R (R Core Team, 2020). Inverse variance method is used for pooling.

**Figure S23**

Model- and meta-level estimates for WEAT (positivity - negativity) comparing companion animals with unappealing wild animals.


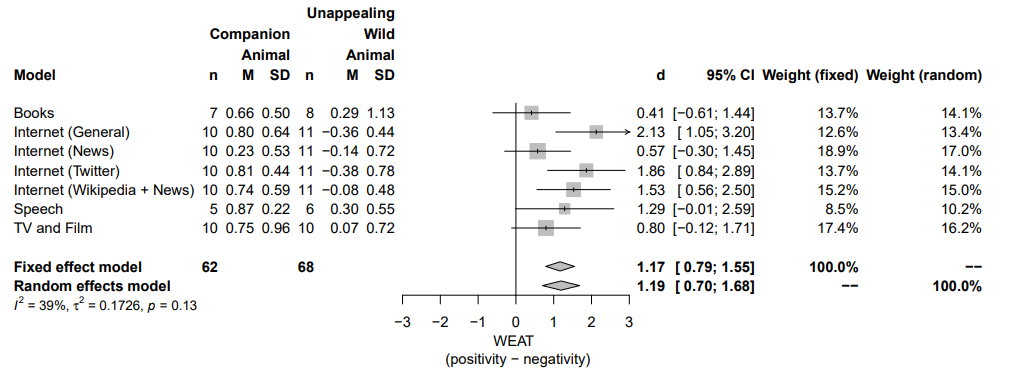


*Note.* Figure depicts means (points), model-level 95% CIs (whiskers), weights (shaded boxes), and meta-level estimates (diamonds). Model is fit via the *meta* (Schwarzer, 2007) package for R (R Core Team, 2020). Inverse variance method is used for pooling.
